# A “SHort course Accelerated RadiatiON therapy” (SHARON) During and Beyond the COVID-19 Pandemic

**DOI:** 10.3389/fonc.2022.823445

**Published:** 2022-02-23

**Authors:** Alessio G. Morganti, Gabriella Macchia, Francesco Cellini, Francesco Deodato, Alice Zamagni, Giambattista Siepe, Milly Buwenge

**Affiliations:** ^1^ Radiation Oncology, Istituti di Ricovero e Cura a Carattere Scientifico (IRCCS) Azienda Ospedaliero - Universitaria di Bologna, Bologna, Italy; ^2^ Dipartimento di Medicina Specialistica Diagnostica e Sperimentale (DIMES), Alma Mater Studiorum - Bologna University, Bologna, Italy; ^3^ Gemelli Molise Hospital, Radiotherapy Unit, Università Cattolica del Sacro Cuore, Campobasso, Italy; ^4^ Dipartimento di Diagnostica per Immagini, Radioterapia Oncologica ed Ematologia, Fondazione Policlinico Universitario “A. Gemelli” Istituti di Ricovero e Cura a Carattere Scientifico (IRCCS), Rome, Italy; ^5^ Dipartimento Universitario Diagnostica per immagini, Radioterapia Oncologica ed Ematologia, Università Cattolica del Sacro Cuore, Roma, Italy

**Keywords:** radiotherapy, palliation, COVID, clinical management, fractionation

The current pandemic situation posed significant problems for radiotherapy (RT) services. In addition to the need to treat COVID-positive patients, it is important to protect health workers and healthy patients from the infection. Although some restrictions are being removed, it is not sure when the pandemic is actually going to be definitively over. Radiation oncologists (ROs) will be forced to face the pandemic for an unknown time interval (1). A recent guideline has been published on the possibility of adapting RT strategies in all settings (2). Particularly along the first months of pandemic spread, hypofractionated RT schedules adequately managing different clinical settings have been proposed to reduce the number of interactions and contacts in hospitals (for both patients–patients and patients–RT personnel), while delivering effective treatments (3–5). Only few were specifically dedicated to palliative RT or particularly oriented to relevant palliative presentations (e.g., bone metastases) (6). With the aim of decreasing hospital contacts, it has been proposed to omit, or delay, or modify the usual prescribed RT regimens (6), more often for palliative settings. However, in the field of palliative RT any omission and delay can dramatically worsen patients’ quality of life. In fact, the proposal to omit palliative radiotherapy during the COVID-19 pandemic has not been widely accepted, with some authors being worried by its clinical and ethical implications (7, 8). We would like to draw attention to a RT regimen tested in different settings. This scheme of SHort course Accelerated RadiatiON therapy: “SHARON” allows to complete a palliative RT course in four sessions and in only 2 days, using a double-daily fractionation.

The obvious advantage of this regime is the possibility of leaving home, in this lockdown phase, for only 2 days. In addition, reducing the RT treatment time also reduces the duration of patient exposure to the hospital environment and the overcrowding of RT departments. In particular, in this way the duration of contacts between potentially positive patients, even if asymptomatic, with health professionals is reduced. The contagion of these latter, especially if affecting a significant number of individuals, could produce a prolonged interruption of all RT treatments with catastrophic consequences in terms of RT effectiveness and in particular of probability of cure. Similar approaches based on acceleration of the treatment course have also been published and applied in clinical practice (9, 10); therefore, the proposed schedule, although innovative, is not strictly experimental.

The experimentation of the SHARON scheme began with a phase I study on brain metastases (11), followed by subsequent trials and analyses (12–20). In particular, the SHARON schedule was extensively analyzed in multiple settings for symptomatic clinical presentations: brain metastases, thoracic, esophageal, pelvic, and “head and neck” lesions, and complicated bone metastases.

**Table 1** summarizes the results of papers published in extenso on the SHARON regimen. Briefly, with a median follow-up ranging between 4 and 7.4 months, reported over the available reports (including: Phase I, Phase II, and Phase I–II trials), the overall symptom response rate ranged between 56% and 96.5% among the different anatomical areas and for the respective symptoms detailed in the trials (e.g., pain, bleeding, dysphagia). Of note, as reported in **Table 1**, the rate of acute toxicity equal or superior to Grade 3 (according to the Radiation Therapy Oncology Group - RTOG - Scale) is inferior to 7% and mostly does account for 0%–2% in all the Phase I and II studies; moreover, the late toxicity is extremely low. As it is evident, the results in terms of tolerability and efficacy in controlling symptoms were positive.

**Table 1:** Main results of the SHARON studies.

Author/Year	Setting	Study Design	Total Dose (4 Fractions/2 days)	No. of Patients	Median Follow-up (months)	Efficacy	Toxicity (Radiation Therapy Oncology Group - RTOG - Scale)	Notes
Caravatta L et al. (11)	Multiple (≥ 3) brain metastases, RPA ≥ 2	Phase I	12–18 Gy	49	5	Overall symptom response rate, 76.2%	Acute G ≥ 3: 2.0% Late *^a^*: 0.0%	Treatment is well tolerated up to 18 Gy
Caravatta L et al. (13)	Multiple (≥ 3) brain metastases, ECOG PS 2	Phase II	18 Gy	50	6	Overall symptom response rate: 63.0%	Acute G3: 6% Late G ≥2: 0.0%	Median OS: 7 months
Farina E et al. (15)	Age > 80 ys; several sites of advanced or metastatic cancer	Pooled analysis of phase II trials	14–20 Gy	48	5.5	Overall symptom response rate: 91.7%	Acute and late G4: 0.0%	
Farina E et al. (15)	Advanced H&N cancers	Phase I–II	14–20 Gy	48	4	Overall symptom response rate: 82.7% (at 20 Gy)	Acute G3: 2.1% Late *^a^*: 0.0%	Treatment is well tolerated up to 20 Gy
Capuccini J et al. (17)	Complicated bone metastases	Phase I–II	16–20 Gy	45	4	Overall symptom response rate: 84.0% (at 20 Gy)	Acute G3: 3.2% Late *^a^*: 0.0%	Treatment is well tolerated up to 20 Gy
Farina E et al. (15)	Thoracic advanced or metastatic tumors	Phase I–II	16–20 Gy	54	5	Overall symptom response rate: 96.5% (at 20 Gy)	Acute G≥3: 1.9% Late *^a^*: 0.0%	Treatment is well tolerated up to 20 Gy
Farina E et al. (16)	Pelvic advancer or metastatic tumors	Phase II	18 Gy	25	6	Overall symptom response rate: 96.0%	Acute G≥3: 0.0 Late *^a^*: 0.0%	
Zamagni A et al. (14)	Multiple bone metastases in lumbar spine plus bony pelvis plus femurs	Phase I	13–15 Gy	25	7.4	Overall pain response: 76.0%	Acute G≥3: 0.0 Late G1: 8.0%	Treatment is well tolerated up to 15 Gy
Deressa BT et al. (12)	Esophageal advanced and symptomatic cancer	Phase II	12 Gy	17	7	Overall symptom response rate: 56%–76% *^b^*	Acute G≥3: 0.0 Late *^a^*: 0.0%	

ECOG PS, Eastern Cooperative Oncology Group performance status; RTOG, Radiation Therapy Oncology Group; G, grade; H&N, head and neck; No., number; OS, overall survival; RPA, recursive partitioning analysis; ys: years.

a Any grade.

b Depending by the specific symptom.

Furthermore, this regimen clearly resulted to be feasible and effective also in the elderly patient population (19) for whom the reduction of the RT duration can be particularly useful. In addition, the SHARON treatment has proved feasible and effective even in a trial conducted in a developing country (12), where the possibility of reducing waiting lists is a further benefit for the aforementioned advantages. Thus, the concern possibly delivered by administration of multiple daily RT sessions is very much mitigated by evidence bases. A single note of caution is that the 3D-conformational technique was used in all studies of the SHARON project, except in one case (12). Finally, the logistical advantage in the use of palliative regimes of the same timing in different settings must be underlined. In fact, this allows the assignment of homogeneous slots in the machine times dedicated to symptomatic treatments.

Due to its efficacy, safety, and easy handling, this set of regimens has been recently included into a guideline for palliative RT indications oriented to patients dealing in complex logistics scenarios (including but not limited to COVID-19 pandemic scenarios) (21). Furthermore, seven clinical controlled randomized trials are ongoing to investigate in multiple metastatic symptomatic presentations (brain, “head and neck,” thoracic, esophageal, abdominal, pelvic, and complicated bone metastases) if the proposed regimen is equally effective to the more commonly applied standard regimen of delivering 30 Gy in 10 fractions (3 Gy each) over 2 weeks; the pending result of these trials will even potentially suggest to include the “Sharon” RT schedule into the routine clinical palliative RT practice beyond the proposed scenario of management for the COVID-19 pandemic.

In summary, during COVID-19 the highlighted “Sharon” RT schedule prevents the risk to avoid palliative RT when needed, offers the advantage of an optimized logistic, reduces patient-to-health professional interactions, minimizes patients’ hospitalization, and offers an equally efficient and isotoxic clinical outcome.

Therefore, we would like to suggest to the community of radiation oncologists to adopt or at least to test this regimen when palliative RT delivered in a single fraction is not considered. Therefore, this regimen could reasonably be used with lower doses (14–16 Gy) if a 2D-conventional technique is employed.

## Author Contributions

AM: concept of RT schedule. GM: adaptation of RT Schedule to different pathologies. FC: paper supervision. FD: trial supervision, paper revision. AZ: paper drafting. GS: paper preparation. MB: ongoing project supervision. All authors contributed to the article and approved the submitted version.

## Conflict of Interest

The authors declare that the research was conducted in the absence of any commercial or financial relationships that could be construed as a potential conflict of interest.

## Publisher’s Note

All claims expressed in this article are solely those of the authors and do not necessarily represent those of their affiliated organizations, or those of the publisher, the editors and the reviewers. Any product that may be evaluated in this article, or claim that may be made by its manufacturer, is not guaranteed or endorsed by the publisher.
